# 375. Patient Safety Measures to Identify Excess Duration and Inappropriately Broad Antimicrobial Use in Uncomplicated Community-Acquired Pneumonia

**DOI:** 10.1093/ofid/ofaf695.122

**Published:** 2026-01-11

**Authors:** Andrea T White, Matthew B Goetz, Makoto M Jones, Claire Ciarkowski, Melinda M Neuhauser, Arjun Srinivasan, David Ratz, Lauren Dutcher, Mingyuan Zhang, Brittany Kent, Lindsay A Petty, Tejal N Gandhi, Jennifer Horowitz, Ashwin Gupta, Valerie M Vaughn

**Affiliations:** University of Utah, Salt Lake City, Utah; VA Greater Los Angeles Healthcare System, Los Angeles, California; Veterans Affairs, Salt Lake City, Utah; University of Utah School of Medicine, Salt Lake City, Utah; Division of Healthcare Quality Promotion, Centers for Disease Control and Prevention,, Atlanta, GA; Centers for Disease Control and Prevention, Atlanta, GA; VA Ann Arbor Health System, Ann Arbor, Michigan; University of Pennsylvania Perelman School of Medicine, Philadelphia, Pennsylvania; University of Utah, Salt Lake City, Utah; University of utah, Salt Lake City, Utah; University of Michigan, Ann Arbor, Michigan; Michigan Medicine, Ann Arbor, Michigan; Michigan Medicine, Ann Arbor, Michigan; Michigan Medicine, Ann Arbor, Michigan; University of Utah, Salt Lake City, Utah

## Abstract

**Background:**

The National Healthcare Safety Network (NHSN) antimicrobial use (AU) module provides a risk-adjusted benchmark of antibiotic use but does not assess appropriateness. To help inform stewardship and reduce antibiotic-related harm, we developed two electronic clinical quality measures (eCQMs) of appropriate antibiotic duration and empiric selection for community-acquired pneumonia (CAP), the most common indication for inpatient antibiotic use and overuse.

**Figure 1.**
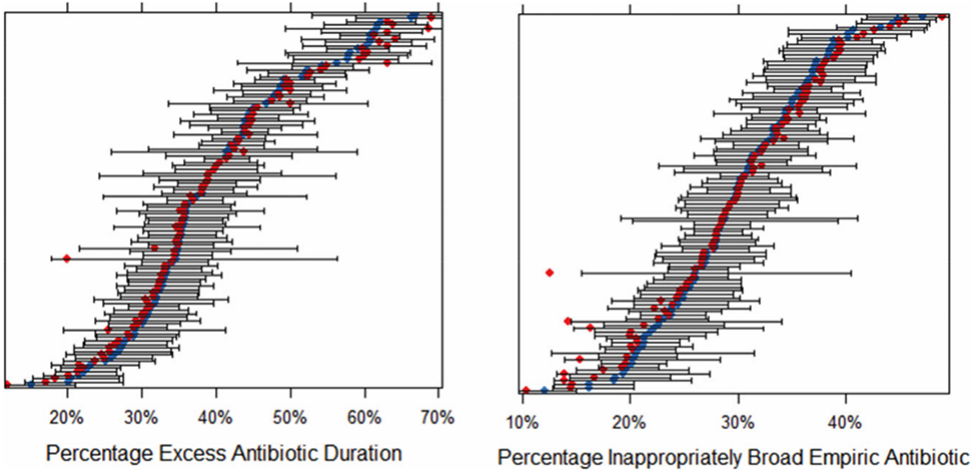
Percentage of hospitalized CAP patients who received excess antibiotic duration (left panel) and inappropriately broad empiric antibiotic therapy (right panel) as defined by our proposed eCQMs across 109 VA health care systems (1/1/2022-6/30/2024) Each row of the above caterpillar plot represents a single VA health care system with the raw (unadjusted) percentage in red and the estimated percentage from a mixed effects logistic model with healthcare system random effects shown in blue (the bar represents the 95% confidence interval). Model-predicted estimates are shrunk towards the mean, meaning that the smaller the number of samples from a particular healthcare system, the more the estimate for that system is pulled toward the global mean.

**Figure 2.**
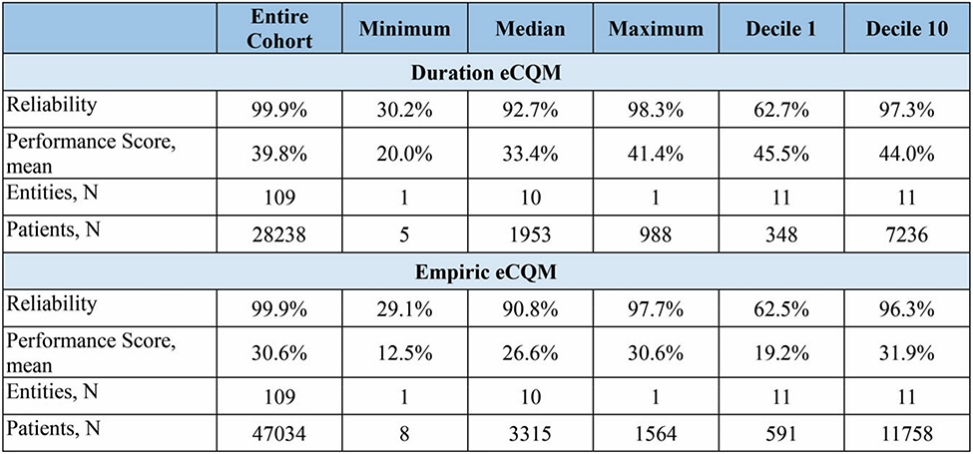
Accountable Entity-Level Reliability Testing Results for Duration and Empiric Therapy eCQMs Calculations were derived from clinical data from 109 Veterans Affairs (VA) healthcare systems between Jan 2022 and June 2024 (n=28,238 and n=47,034 hospitalized pneumonia patients for duration and empiric measures, respectively).

**Methods:**

We developed 2 eCQMs for hospitalized adults with uncomplicated CAP to quantify: a) excess antibiotic duration (i.e., ≥ 7 days in a patient eligible for 5 days) and b) inappropriately broad empiric therapy (i.e., targeting methicillin-resistant *Staphylococcus aureus* or *Pseudomonas aeruginosa* without risk factors for drug resistance). The eCQMs were derived from chart review measures used by the Michigan Hospital Medicine Safety Consortium and further developed and validated using Epic data from two academic medical centers and Computerized Patient Record System data from 109 Veterans Affairs (VA) hospitals. From these data sources, we assessed reliability (signal-to-noise analysis using beta-binomial regression), validity (sensitivity/specificity), and feasibility (availability of structured data in electronic records) of both eCQMs.

**Figure 3.**
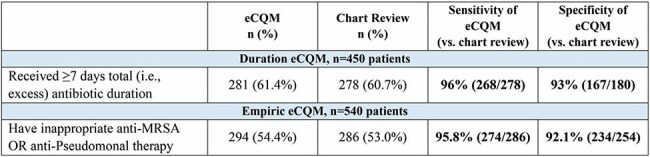
Sensitivity/Specificity of eCQMs Comparing eCQM and Chart Review Abbreviations: eCQM, electronic clinical quality measure; ICU, intensive care unit; MRSA, methicillin-resistant Staphylococcus aureus; IV, intravenous

**Figure 4.**
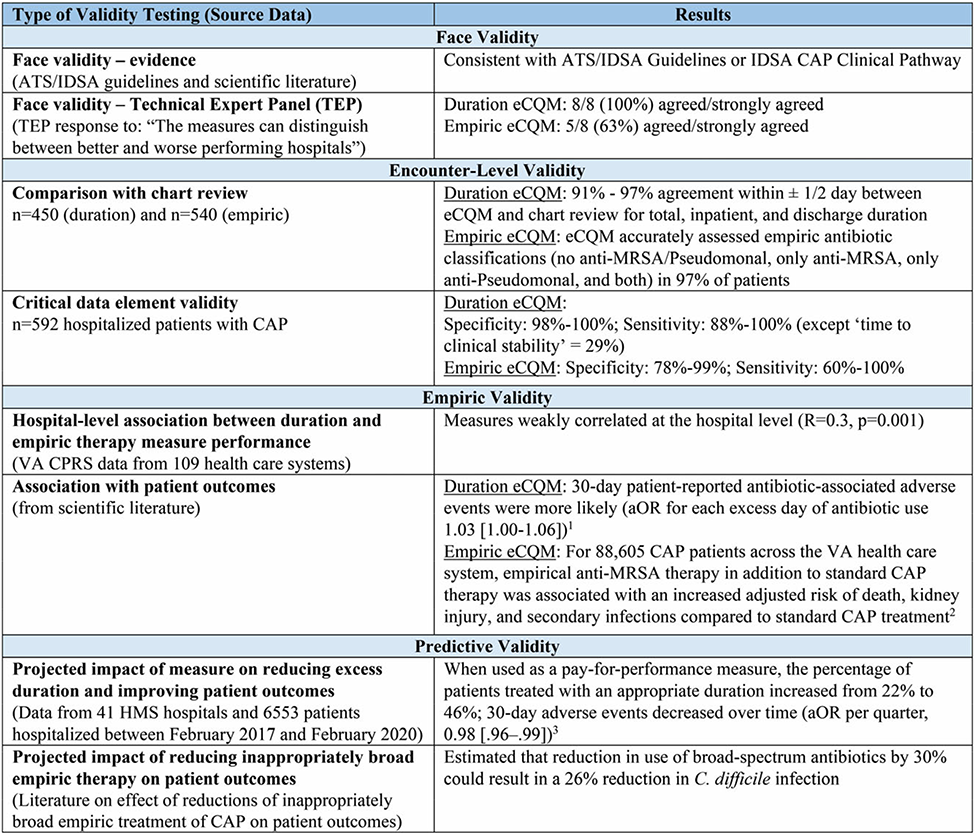
Validity Testing Types, Data Sources, and Results for eCQMs for Duration and Empiric Therapy Abbreviations: ATS, American Thoracic Society; IDSA, Infectious Diseases Society of America; CAP, community-acquired pneumonia; eCQM, electronic clinical quality measure; MRSA, methicillin-resistant Staphylococcus aureus; VA, Veteran’s Administration; CPRS, Computerized Patient Records System; aOR, adjusted odds ratio

1 Vaughn VM, Flanders SA, Snyder A, et al. Excess Antibiotic Treatment Duration and Adverse Events in Patients Hospitalized with Pneumonia: A Multihospital Cohort Study. Annals of internal medicine. Aug 6 2019;171(3):153-163. doi:10.7326/M18-3640

2 Jones BE, Ying J, Stevens V, et al. Empirical Anti-MRSA vs Standard Antibiotic Therapy and Risk of 30-Day Mortality in Patients Hospitalized for Pneumonia. JAMA Intern Med. Apr 1 2020;180(4):552-560. doi:10.1001/jamainternmed.2019.7495

3 Vaughn VM, Gandhi TN, Hofer TP, et al. A Statewide Collaborative Quality Initiative to Improve Antibiotic Duration and Outcomes in Patients Hospitalized With Uncomplicated Community-Acquired Pneumonia. Clin Infect Dis. Aug 31 2022;75(3):460-467. doi:10.1093/cid/ciab950

**Results:**

When we applied the eCQMs to our 3 data cohorts, the mean percentage of patients with excess duration and inappropriate empiric antibiotic use ranged from 39.8% to 54.5% and 16.6% to 55.9%, respectively, with the performance gap across the VA cohort shown in Figure 1. Across all 109 VA hospitals the accountable entity-level reliability for both eCQMs was >99.9% (details in Figure 2). Compared to chart review, the sensitivity and specificity for both eCQMs was high (92%-96%, Figure 3). Additional validity results are shown in Figure 4. Feasibility was maximized by using only data in structured fields.

**Conclusion:**

We developed two eCQMs related to CAP that had high reliability, validity, and feasibility. Both eCQMs were endorsed “with conditions” by Battelle and are being submitted to the CMS “Measures Under Consideration” list. If broadly used, these measures could help antibiotic stewards measure and improve antibiotic selection and duration for CAP across the US.

**Disclosures:**

All Authors: No reported disclosures

